# Evaluation of Pharmacy-Led Post-Discharge Follow-Up on Transitional Care Management in a Tertiary Academic Hospital: An Observational Study

**DOI:** 10.7759/cureus.43477

**Published:** 2023-08-14

**Authors:** Abdulhamid Althagafi, Mohannad Alshibani, Samah Alshehri, Abdulrahman Alqarni, Mohammed Baharith, Salih Alqurashi

**Affiliations:** 1 Pharmacy Practice, King Abdulaziz University, Jeddah, SAU; 2 Clinical Pharmacy, King Abdulaziz University, Jeddah, SAU

**Keywords:** pharmacy follow-up, patient education, hospital discharge, high-medication-risk patients, medication-related problems

## Abstract

Introduction: The administration of multiple medications and complex drug regimens has increased medication-related problems (MRPs) and associated factors. MRPs can occur at any stage of the medication process and are common after hospital discharge. Understanding and managing these problems are crucial for promoting safe and effective medication use.

Objective: This study aimed to evaluate the prevalence of MRPs among post-discharge patients with high medication risk in the academic tertiary care hospital of King Abdulaziz University (KAUH) in Jeddah, Saudi Arabia.

Methods: A prospective cross-sectional study was conducted, and data were collected through phone calls to discharged patients using validated questions. MRPs were identified based on the classification of the Pharmaceutical Care Network Europe (PCNE), and data analysis was performed using descriptive statistics.

Results: Out of 287 screened participants, 201 fulfilled the inclusion criteria. The prevalence of MRPs among high-medication-risk patients after hospital discharge was substantial, with 519 MRPs identified. The most common types of MRPs were the need for medication information, untreated symptoms or indications, and nonadherence.

Conclusion: The most prevalent MRPs among patients in our hospital were the need for education and untreated symptoms or indications. Future studies should investigate MRPs in larger samples and explore interventions by pharmacists.

## Introduction

The administration of multiple medications and the increasing number of available drugs, as well as more complex drug regimens, has led to more medication-related problems (MRPs) and associated factors [1]. MRPs are generally defined as events or circumstances involving drug therapy that actually interfere with or probably interfere with planned patient-related outcomes. The Pharmaceutical Care Network Europe (PCNE) further categorizes MRPs based on problem and cause domains that include patient-related factors, drug-drug interactions, treatment duration, and others [2]. MRPs are common after hospital discharge due to deficient patient or caregiver education, poor communication between healthcare providers, and inadequate follow-up post-discharge [3].

The incidence of MRPs ranges from 18.4% two weeks post-discharge up to 37.5% one month post-discharge [4]. MRPs can occur at any stage of the medication process, including prescription, dispensing, administration, and monitoring. Understanding these problems is essential to promote safe and effective medication use [5]. Managing patients with high medication risk after hospital discharge is a crucial component of a successful discharge process that ensures patient safety and prevents hospital readmissions [6]. A close evaluation after discharge is often required to identify the most common MRPs and develop a strategy to reduce them [7]. MRPs often result from patients’ drug-related concerns that are not discovered or addressed and may lead to clinical complications if they are not identified and managed. They may also lead to a higher risk of death by increasing the number of visits to ambulatory care units or the length of hospital stays [8]. More people have died from inappropriate drug treatment than from car accidents, breast cancer, or acquired immune deficiency syndrome combined. Therefore, it is important to identify and address MRPs through medication therapy management, which involves optimizing medication use to achieve desired therapeutic outcomes and minimize the risk of adverse events [9].

Studies have demonstrated that post-discharge telephone follow-up is a well-established method for exchanging information with patients and reducing readmission rates [10-12]. MRP awareness is needed to provide better patient care and may help in identifying, resolving, and preventing potential MRPs. Despite the high prevalence of MRPs, no adequate studies have been conducted in Saudi Arabia. Therefore, the objective of our study was to evaluate the prevalence of MRPs identified in an academic tertiary care hospital among post-discharge patients with high medication risk in the country. The findings of this study may also impact the development of appropriate plans, policies, and intervention programs for the management and prevention of MRPs. Hence, this might enhance the quality of care provided to hospital patients.

## Materials and methods

This prospective cross-sectional study was conducted in an academic tertiary care hospital of King Abdulaziz University (KAUH) in Jeddah, Saudi Arabia. From January 2022 through August 2022, data collection was performed by well-trained pharmacy interns using questions adopted from previous studies and validated on 13 patients [13]. Interns contacted discharged patients within one week from the day of discharge to follow-up. Phone calls were conducted using the hospital’s official landline to confirm patient privacy. The MRP evaluation was prepared based on the classification of the PCNE and focused on problem and cause domains. All MRPs were identified by three intern pharmacists and confirmed by two clinical pharmacists. Then, collected data were checked for drug-drug interactions using updated drug information software, such as Lexicomp and Micromedex databases, to determine the presence of MRPs. Using the hospital’s electronic health records (EHRs), data on patient demographics, past medical history, medication history, and current medications were recorded for each patient. Patients are eligible for screening if they are 18 years of age or older and treated with a high medication risk. Patients were included if they were discharged from the hospital, responded to phone calls within one week of discharge, and performed a complete phone call medication reconciliation. Patients were excluded if there was any missing information, a language barrier, or an inability to contact the patient after three attempts on three different days.

Definitions

High-risk-medication patients were defined as those with polypharmacy, oral anticoagulants, anti-infectives, insulins, and narcotics.

Medication discrepancies were defined as any difference between the discharge medication list and medications the patient reported taking.

Study outcomes and data analysis

The objective of our study was to evaluate the prevalence of MRPs identified among high-medication-risk patients after hospital discharge. Descriptive statistics were used to summarize the data, including the mean standard deviation (SD) for continuous data and the frequency (in percentage) for categorical data. The data were analyzed using IBM SPSS Statistics for Windows, Version 28 (released 2021; IBM Corp., Armonk, New York, United States).

Ethics

The study was approved by the ethical review board of KAUH (approval number: 561-21, November 22, 2021). Approval was granted for this study (404-21) by the institutional review board of KAUH on August 10, 2021. Due to the nature of the current study, all data were fully anonymized before accessing.

## Results

Characteristics of the participants

**Figure 1:**
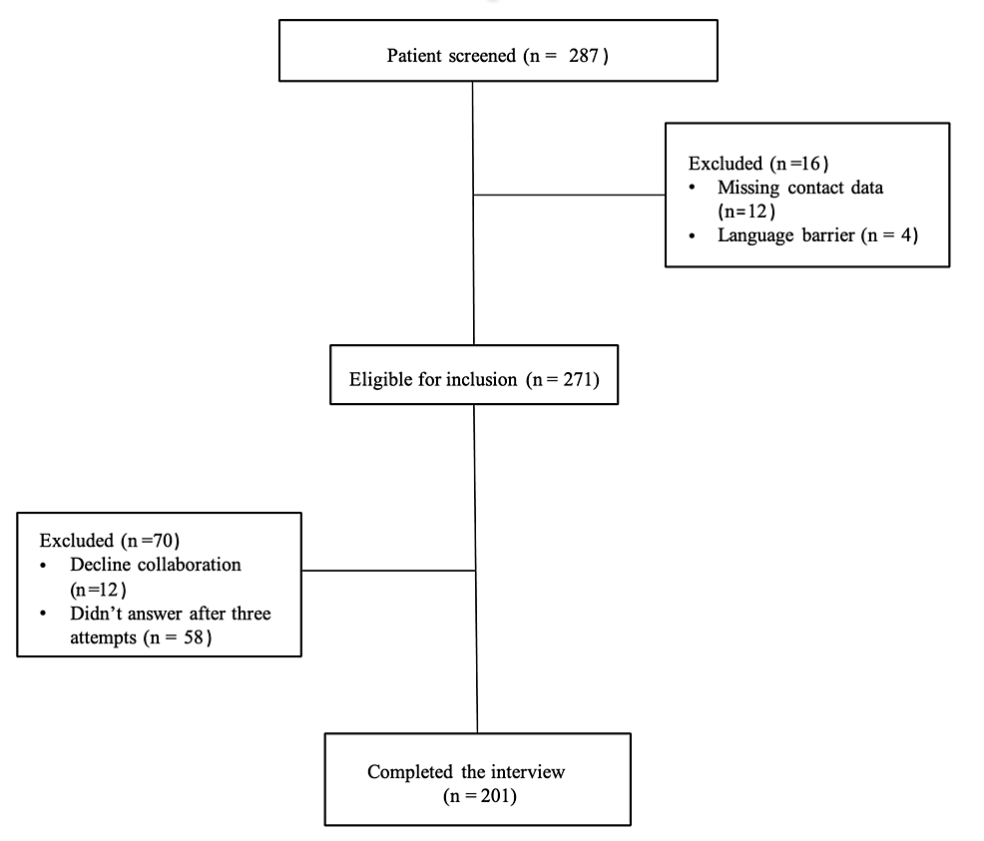
Patient inclusion flowchart

Out of the 287 participants who were screened (Figure 1), 201 (58.7%) fulfilled the inclusion criteria and were included in the study. Regarding participants’ gender, male gender constituted 55.2% of the total respondents, and 44.8% were female. The mean ± SD age of the participants was 57 ±15 years. The majority of the participants, 115 (57.2%), were Saudi nationals. The educational status of the study population showed that 52 (25.8%) had attained a university education. The mean duration of the call was 7.51 minutes per patient. The patients identified taking the following medications: 179 (45%) took more than five medications, 116 (29.14%) were on anticoagulant therapy, 65 (16.33%) were on insulin therapy, 28 (7.03%) were on anti-infectives, and 10 (2.5%) were on narcotics (Table 1).

**Table 1:** Baseline characteristics of the study participants

Baseline characteristics	Value
Age, mean (± SD)	57.7 (15.3)
Female, n (%)	90 (44.8)
Nationality, n (%)	
Saudi	115 (57.2)
Non-Saudi	86 (42.8)
Education, n (%)	
Pre-school	32 (15.92)
Primary school	47 (23.38)
Intermediate school	23 (11.44)
High school	47 (23.38)
College/university	52 (25.8)
Chronic conditions, n (%)	
Diabetes mellitus	105 (29.41)
Hypertension	104 (29.13)
Congestive heart failure	62 (17.36)
Stroke	10 (2.80)
Dyslipidemia	50 (14)
Others	26 (7)
Duration of the call, mean (± SD)	7.51 (1.24)
Post-discharge to call time, mean (± SD)	3.43 (3.43)
Medications at discharge, mean (± SD)	7.25 (2.80)
High-risk medication use, n (%)	
More than five medications	179 (45)
Anticoagulants	116 (29.14)
Insulin	65 (16.33)
Anti-infectives	28 (7.03)
Narcotics	10 (2.5)

Types and frequencies of MRPs

A total of 519 MRPs were identified. The most common type of MRP was the need for medication information (51.93%), followed by untreated symptoms or indications (17.53%), nonadherence (9.44%), failure to receive medication (8.28%), medication discrepancy (5.58%), medication without indication (4%), therapeutic duplication (1.9%), and drug-drug interactions (1.34%; Table 2).

**Table 2:** Primary outcomes and their associated values

Primary outcome	Value, n (%)
Information about medications	269 (52)
Indication	44 (8)
Common side effects	82 (16)
Frequency	23 (4)
Duration	34 (7)
Other information	86 (17)
Untreated symptoms or indications	91 (17.53)
Nonadherence	49 (9.44)
Failure to receive medication	43 (8.28)
Medication discrepancy	39 (7.48)
Difference between discharge and reported medication	29 (5.58)
Therapeutic duplication	10 (1.9)
Medication without indication	21 (4)
Drug-drug interactions	7 (1.34)

## Discussion

The major finding of our study was the prevalence of MRPs identified among high-medication-risk patients after hospital discharge. This prospective cross-sectional study was conducted in an academic tertiary care hospital of KAUH in Jeddah, Saudi Arabia, and aimed to evaluate the prevalence of MRPs and compare our results with previously published studies in the field. The prevalence of MRPs identified in our study population was substantial, with a total of 519 MRPs identified among the high-medication-risk patients. This highlights the significant burden of medication management issues in this population, which could potentially lead to adverse drug events, suboptimal treatment outcomes, and increased healthcare utilization. Identifying and addressing MRPs are crucial to optimize patient safety and ensure effective and appropriate medication use.

The most common type of MRP identified in our study was the need for medication information, accounting for 51.93% of the MRPs. This finding emphasizes the importance of patient education and counseling regarding their medications, including proper usage, potential side effects, and adherence. Providing comprehensive medication information can empower patients to actively participate in their own care and make informed decisions about their treatments. Untreated symptoms or indications were the second most common type of MRP, representing 17.53% of the identified MRPs. This finding suggests that there is a need for improved recognition and management of symptoms and indications, both by healthcare providers and patients themselves. It underscores the importance of effective communication between healthcare professionals and patients to ensure that symptoms are properly assessed, and appropriate interventions are implemented.

Nonadherence was another prevalent MRP, accounting for 9.44% of the identified MRPs. Nonadherence to medication regimens is a well-known challenge in healthcare, leading to treatment failure, disease progression, and increased healthcare costs. Our findings highlight the need for interventions aimed at improving medication adherence, such as patient education, reminders, simplified dosing regimens, and involvement of caregivers or family members in medication management. Failure to receive medication, medication discrepancy, medication without indication, therapeutic duplication, and drug-drug interactions were identified as less frequent types of MRPs in our study. Although these types of MRPs were less prevalent, they are still important to address, as they can have significant implications for patient safety and treatment outcomes. Strategies, such as medication reconciliation processes, electronic prescribing systems with built-in checks, and interprofessional collaboration, can help mitigate these issues.

Comparing our results with previously published articles in the field, several similarities and consistencies can be observed. Similar to our study, previous studies have employed prospective cross-sectional designs to assess MRPs in high-medication-risk patients after hospital discharge by phone calls [14,5]. This design allows for efficient data collection at a specific time point and provides valuable insights into the prevalence and types of MRPs in this population.

Our study findings regarding the prevalence and types of MRPs align with existing literature. Similar to previous research, we found that the need for medication information, untreated symptoms or indications, and nonadherence were among the most common MRPs, and our study findings are consistent with those of a previous study conducted at two acute hospitals located in Buckingham, which found that the most frequent MRPs were for medication information (74%), followed by recommending adherence issues (55.6%) [13]. Similarly, a study conducted in a teaching hospital in the Netherlands showed that the information needed about medications was the most common type of MRP identified [14]. Furthermore, a study conducted in Ethiopia reported that untreated indications and symptoms were the most common MRPs encountered by 32.6% [1]. In addition, the finding is also close to that of a prospective study that showed that the most common type of MRP was non-adherence, followed by medication access issues [5]. By contrast, a prospective cross-sectional study conducted on 225 patients showed that the most frequently encountered MRP was drug-drug interactions (48%) [15]. The discrepancy may be due to the difference in the method used to assess and classify MRPs.

In our study, the PCNE classification of MRPs was used. Furthermore, our study was conducted on one-week post-discharge. These consistent findings underscore the persistent challenges faced by high-medication-risk patients in managing their medications after hospital discharge. They also highlight the need for targeted interventions and strategies to address these MRPs and improve patient outcomes.

Despite the valuable insights provided by our study, it is important to acknowledge its limitations. First, our study was conducted in a single academic tertiary care hospital, which may limit the generalizability of the findings to other healthcare settings. Future research should consider multi-center studies involving diverse patient populations and healthcare facilities to enhance the external validity of the results. Second, the data collection relied on self-reported information provided by patients during phone calls, which may introduce recall bias or incomplete reporting. While we mitigated this limitation by involving well-trained pharmacy interns and using validated questions, objective measures, such as medication reconciliation records, could have provided additional accuracy and reliability.

Furthermore, the study's cross-sectional design limited our ability to establish causal relationships between MRPs and patient outcomes. Longitudinal studies that follow patients over time can provide more robust evidence in this regard. In addition, the study focused on high-medication-risk patients and did not include a control group of low-medication-risk patients for comparison. Including a control group would have allowed for a better understanding of the differences in MRPs between high- and low-medication-risk populations.

## Conclusions

Complex drug regimens lead to MRPs, notably after hospital discharge. This study focused on high-medication-risk patients, revealing significant MRPs, such as knowledge gaps in medications untreated symptoms and nonadherence. These align with prior findings, highlighting the importance of patient education and symptom control. The study highlights the need for tailored interventions to improve post-discharge medication management. Addressing MRPs enhances patient safety and outcomes, ultimately elevating care quality for high-medication-risk post-discharge patients.
